# Genes, proteins and other networks regulating somatic embryogenesis in plants

**DOI:** 10.1186/s43141-020-00047-5

**Published:** 2020-07-13

**Authors:** Basit Gulzar, A. Mujib, Moien Qadir Malik, Rukaya Sayeed, Jyoti Mamgain, Bushra Ejaz

**Affiliations:** Department of Botany, Jamia Hamdard (Hamdard University), New Delhi, 110062 India

**Keywords:** Auxin and cytokinin signaling, Plant growth regulators, *SERK* gene, Stress, Somatic embryo-specific proteins, Transcription factors

## Abstract

**Background:**

Somatic embryogenesis (SE) is an intricate molecular and biochemical process principally based on cellular totipotency and a model in studying plant development. In this unique embryo-forming process, the vegetative cells acquire embryogenic competence under cellular stress conditions. The stress caused by plant growth regulators (PGRs), nutrient, oxygenic, or other signaling elements makes cellular reprogramming and transforms vegetative cells into embryos through activation/deactivation of a myriad of genes and transcriptional networks. Hundreds of genes have been directly linked to zygotic and somatic embryogeneses; some of them like *SOMATIC EMBRYOGENESIS LIKE RECEPTOR KINASE* (*SERK*), *LEAFY COTYLEDON* (*LEC*), *BABYBOOM* (*BBM*), and *AGAMOUS-LIKE 15* (*AGL15*) are very important and are part of molecular network.

**Main text (observation):**

This article reviews various genes/orthologs isolated from different plants; encoded proteins and their possible role in regulating somatic embryogenesis of plants have been discussed. The role of SERK in regulating embryogenesis is also summarized. Different SE-related proteins identified through LC–MS at various stages of embryogenesis are also described; a few proteins like 14-3-3, chitinase, and LEA are used as potential SE markers. These networks are interconnected in a complicated manner, posing challenges for their complete elucidation.

**Conclusions:**

The various gene networks and factors controlling somatic embryogenesis have been discussed and presented. The roles of stress, PGRs, and other signaling elements have been discussed. In the last two-to-three decades’ progress, the challenges ahead and its future applications in various fields of research have been highlighted. The review also presents the need of high throughput, innovative techniques, and sensitive instruments in unraveling the mystery of SE.

## Background

Somatic embryogenesis (SE), the intricate multi-step process nowadays holds prime importance in tissue culture methodology, made big leaps ever since its first report in mid twentieth century [144]. This technique unveils diverse areas where its application is indispensible and provides significant insights in pathways and mechanisms underlying plant development. It is yet another way of mass propagation of plants vegetatively [32, 42]. The regeneration of a complete plant from a single or group of somatic cells is always remaining as the fundamental importance of SE [54]. The technique includes plant regeneration from cells that are already differentiated [62]. Hence, SE is a unique potentiality of plant cells and is triggered with acquired embryonic potential [75]. This paradigm shift occurs after reprogramming of developmental processes, enabling the cells to attain embryogenic competence [100]. The differentiated cells under plant growth regulator (PGR) treatments undergo several morphogenetic changes and attain embryogenic competence [75, 101, 102]. Similarly, the pre-embryogenic determined cells (PEDC) present in explant are committed to produce embryos and enter embryogenesis process under the influence of PGRs and other favorable conditions [75].

**Fig. 1:**
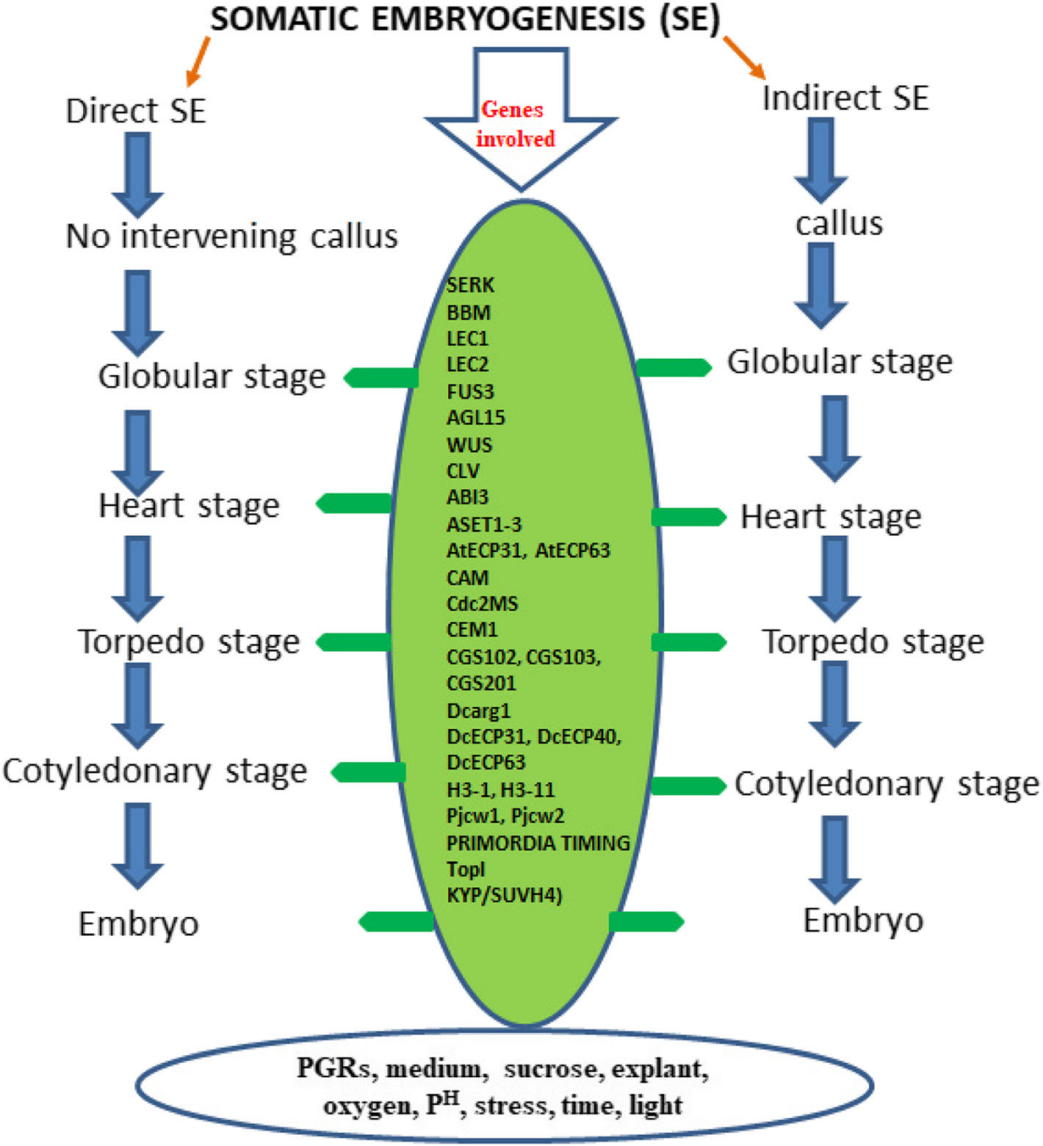
*Two different pathways of SE in dicots (i.e., direct and indirect SE), different (i.e., globular, heart, torpedo and cotyledonary) stages of embryos, factors affecting SE are kept at bottom in oval, and one central green oval shows some genes involved in SE. SERK1-5****(****SOMATIC EMBRYO RECEPTOR KINASE 1-5****),****LEC1, LEC2****(****LEAFY COTYLEDON 1,2****),****BBM****(****BABY BOOM****),****FUS3****(****FUSCA 3****),****ABI3(ABA INSENSITIVE 3****),****AGL15****(****AGAMOUS LIKE 15****),****ASET1-3****(****Alfalfa SE-specific transcripts****),****AtECP31****(****Arabidopsis thaliana Embryogenic31****),****AtECP63****(****Arabidopsis thaliana Embryogenic63 cell proteins)****,****CaM genes****(****Calmodulin genes****),****Cdc2MS****(****Cell division cycle****),****CEM1****(****elongation factor-1α****),****CGS102, CGS103, CGS201****(****Carrot glutamine synthetase****),****Dcarg1****(****Daucas carrotaauxin regulated gene****),****SAUR****(****small auxin up-regulated = Pjcw1****,****Top1****(****topoisomerase1****),****DcECP31****,****DcECP40****,****DcECP63****(****Daucus carota embryogenic cell protein****),****H3-1****,****H3-11****(****Histone 3****),****KYP/SUVH4****(****Kryptonite****),****LBD29****(****LATERAL ORGAN BOUNDARIES DOMAIN 29****),****PRC 1(POLYCOMB REPRESSIVE COMPLEX1****)***

The process of SE has various phases like initiation, proliferation, maturation, and conversion [58]. Phase 0 is suggested to have competent single cells giving rise to embryogenic clusters under the influence of PGRs especially auxin [33, 150]. In this phase, different cell clusters acquire the competence to develop embryos. The phase 1 starts by transferring embryogenic cell clusters to an auxin-free medium, and the cell clumps proliferate slowly and do not differentiate [33]. This phase is followed by rapid cell division of cells, giving rise to globular embryos referred to as Phase 2. Embryos of different shapes (heart, torpedo, and others) constitute Phase 3 [33]. Drastic morphological, physiological, and biochemical changes set in during meristem (shoot, root) differentiation [135, 153]. The in vitro microenvironment is very stressful, and this could be osmotic and wounding and have micronutrient supply, desiccation, and PGR stress; and these adverse stresses trigger reprogramming of cellular development [28]. The already differentiated cells dedifferentiate or acquire embryogenic competence, and the entire phenomenon is often governed by hundreds of genes [28, 56, 115]. At different stages of SE, a distinct set of genes activate in developing embryos [64], and these genes regulate steps in switching from one development stage to the other [123]. Chromatin reorganization, the activation and deactivation of one or more genes (Fig. 1), carry out a cascade of activities and are perhaps the reason behind cellular transition. Only a few of these genes have been extensively studied while the other genes’ role in embryogenesis is still a mystery [28].

The embryogenic cell/cells transforming embryos could histologically be distinguished from others by some characteristics like cell wall with cellulose, denser cytoplasm, fragmented vacuole, highly active nucleus with large nucleolus, high nucleus-to-cytoplasm ratio, and low level of heterochromatin [13, 147]. At molecular level, the features of embryogenic tissues have not been comprehensively distinguished because of the usage of the whole explant in expression analysis [13, 147]. Explants possess a variety of cells arranged in a complex fashion, posing problems in molecular marker-based identification of embryogenic cells.

Various embryo stages are present in the process of SE, named after the shape attained by the growing embryo in the course of development (Fig. 1). These stages are globular, heart, torpedo, and cotyledonary in most of the dicot plants, while globular, scutellar, and coleoptilar in monocots, and early immature, pre-cotyledonary, early cotyledonary, and late cotyledonary embryos in conifers [42, 103, 116]. Mikula et al. [98] reported three different morphogenetic stages of somatic embryos in fern—i.e., linear stage (spanning first cell division to several-celled proembryo), early embryonic leaf stage (until the emergence of first leaf), and late embryonic leaf stage (showing the appearance of second leaf). SE is induced either directly in explants or indirectly on callus [157]. In the former, SE occurs without forming any intervening callus, whereas indirect SE is always characterized by the formation of callus. In direct SE, the cells are determined to become embryos shortly after the reprogramming sets in without prior division of cells, while in indirect SE, embryogenic competence is attained comparatively later after formation of callus [115]. In certain cases, the embryogenic competence is often preceded by cell division, and induced embryogenic determined cells (IEDC) are formed by dedifferentiation of differentiated cells which lead to embryogenic development [141, 148]. Induction of SE is very difficult in the older tissue, and it may be of direct or indirect origin, but it is rather difficult to generate embryogenic competent cells from aged tissue as older cells take time to reprogram it [75]. This is perhaps the reason why developmentally older tissues take only the indirect route of embryogenic development [9]. The embryos are induced directly or indirectly on explants called primary somatic embryogenesis, while the formation of embryo on primary embryos is termed as secondary somatic embryogenesis. In this phenomenon, the primary embryo does not convert into a complete plantlet and instead gives rise to many secondary embryos [104]. Somatic embryos are bipolar structures and have no vascular connections with the underlying plant, one of the features distinguishing it from the other plant organs and zygotic embryos [149]. The bipolar structure contains an independent provascular system, and each of the pole has its own meristem [24, 68].

## Somatic embryogenesis incidences and various networks

### Embryogenesis and woody genera

In certain plant groups like woody genera, response is poor in developing callus and embryogenic tissues; the exudation of phenolics and similar other compounds aggravate the problem further [18]. With the growing knowledge and other technological advances, these problems were overcome in many plants, and consequently, many woody plants are now cultured in vitro. But most of the woody plants are still either completely reluctant or respond poorly to treatments for embryogenesis [42]. With the current high demand for woody plants (due to medicinal, aesthetic values, food, fiber, timber, fuel), plant conservation concerns and climate change attract researchers’ attention in unveiling new strategies for rapid, mass propagation of such plants. Marker-assisted breeding, genetic transformation, etc. are also being targeted to improve plant quality [42, 82, 95]. SE is one of the methods being continuously upgraded and renovated to suit plant propagation particularly for those plants that have a long life cycle, produce less/no seeds, and do not reproduce vegetatively. This technique is preferred over the organogenesis because of bipolar embryo that does not need separate treatment for root or shoot induction [159]. The bipolar embryonal axis has both shoot and root ends and is directly grown to complete plants [24]. Various factors govern SE induction and embryo numbers such as plant genotype, type of explants, type and strength of stimulus, and age of tissue (e.g., juvenility) [113]. After acquisition of embryogenic competence, embryo development may not always reach the final stages of plantlet formation [164]. In plants, where embryos developed, a similar developmental pattern was observed for the attainment of other developmental stages. Thus, SE is suitable for forest and other groups of plant propagation, genetic engineering, and cryopreservation of elite germplasm [14, 95, 110].

### Genes regulating vegetative to embryonic (early stage) transition

LAFL network genes [*LEAFY COTYLEDON1*, LEC1/LEC1-LIKE (L1L), ABSCISIC ACID INSENSITIVE 3 (ABI3), *FUSCA3* (*FUS3*), and (*LEC2*)] are involved in the initial steps of direct SE which is not true for indirect SE in *BABYBOOM* (*BBM*)-mediated LAFL [LEC1/LEC1-LIKE (L1L), *ABSCISIC ACID INSENSITIVE 3* (*ABI3*), *FUSCA3* (*FUS3*), and (*LEC2*)] gene expression [10]. Chromatin state of LAFL gene is one of the factors that determine direct or indirect SE. *LEC1/LEC1-LIKE* (*L1L*) and *LEC2* induce direct SE when constitutively overexpressed, while *LEC1* in particular is detected later after embryo appears on the callus surface [44].

### Role of plant growth regulators (PGRs) in embryogenesis network

PGRs play a key role in both zygotic and somatic embryogeneses. Among all PGRs, auxin is most effective in the induction of SE [94, 112, 138]. Once SE is induced, auxin concentration is either to be lowered or completely omitted [117]. Different PGRs, their concentrations and combinations have different effects on the process of SE depending on the plant species. In most species, auxin, cytokinin, abscisic acid (ABA), and jasmonic acid (JA) are the key factors triggering the embryogenic response as these have a regulatory effect on cell cycle, division, and differentiation [29]. Auxin 2,4-dichlorophenoxyacetic acid (2,4-D), either alone or in combination with cytokinins, is used to induce somatic embryo in many plant species using seeds or zygotic embryos as explants [29, 61, 118]. Synthesis of jasmonic acid and abscisic acid (stress-related PGRs) was reported in *Medicago sativa* throughout the process of SE but differentially biosynthesized in different phases of SE. Gibberellins (GAs), usually gibberellic acid (GA_3_), have a repressive role on the induction of SE in some plants as it significantly upregulates gibberellins 2-oxidase (GA2ox6), repressing GA synthesis (Elhiti et al. 2010).

LEAFY COTYLEDON 1 (LEC1) is a key player in abscisic acid (ABA)-mediated expression of *YUCCA10* (*YUC10*) in seedlings [72]. *YUC* mutants (YUC genes are involved in auxin biosynthesis) are less responsive to secondary SE, suggesting that the endogenous auxin is important for this process [151]. Adventitious shoot formation is induced in short auxin exposure while somatic embryo formation in long auxin exposure. This suggests the developmental continuum in somatic embryo and adventitious shoot formation, where critical threshold auxin signaling is crucial in in vitro induction and maintenance of embryo identity [112]. Auxin-mediated plant development involves changes in expression of auxin-responsive genes, encoding a family of transcription factors, AUXIN RESPONSE FACTORs (ARFs). The ARFs regulate the expression of target genes by binding to AUXIN RESPONSE ELEMENT (AuxRE) TCTCTC motif, present in promoters of auxin-responsive genes [150]. The ARFs bind promoters via a B3-type DNA binding domain, specific to plants. Molecular studies of *Arabidopsis thaliana* identified about 22 ARF genes and a pseudogene [86]. Among the different ARFs, ARF5, ARF6, ARF7, ARF8, and ARF19 activate the target gene expression, while ARF1, ARF2, ARF3, ARF4, and ARF9 repress the expression of target genes. Wójcikowska and Gaj [150] observed upregulation of four ARFs (ARF5, ARF6, ARF10, and ARF16) during the inductive phase of SE in *Arabidopsis*, while two ARFs (ARF8 and ARF17) were upregulated in advanced stages. A number of ARFs are being identified in different plants, and intensive research continues in this field to elucidate their role in plant developmental processes.

### Plant genotype, explants, and oxygenation determining embryogenesis

The success in regenerating plant via SE is largely dependent on the genotype of the plant species [27, 65]. Different plant parts respond differently, while cultured in vitro or even different genotypes of a plant behave uniquely/differently. Sané et al. [124] reported that Ahmar and Amsekhsi cultivars were more callogenic than Tijib and Amaside, exhibiting response differences in primary callogenesis in different date palm cultivars. Similarly, woody plants are more recalcitrant in showing responses than the herbaceous groups of plants [18, 65].

Various types of explants are used for generating somatic embryos in different plants. The type and size of explant and plant species significantly influence the process of SE [140]. Kocak and co-workers [79] demonstrated that the leaves and petioles of *Cyclamen persicum* were more responsive compared to the ovule and ovary and took less time to induce callus; in carnation, callus followed by somatic embryos were obtained from petal explants in a number of cultivated varieties [76].

The dissolved oxygen concentration in culture flask has significant influence on the development of somatic embryos. It is observed that the concentration of oxygen in suspension had ostensible effects on the maturation process and the number of embryos [13, 22]. The 50% dissolved oxygen (DO) levels in medium showed maturated embryos with lower numbers, while at 80% DO concentration, opposite response (i.e., higher embryo numbers with less maturity) were noted in *Coffea arabica* [13].

### Somaclonal variation, SE, and genetic integrity

Somaclonal variation (SV) is a phenomenon whereby the variations are manifested among the tissue culture-raised plants, and these variations include both phenotypic and genotypic alterations [99]. The genetic alterations occur spontaneously under stressed microenvironment and can continue to remain for several generations [20]. The changes are heritable and non-heritable containing point mutation, chromosomal deletion, substitution, DNA breakage, and ploidy [97, 154]. The PGR-induced stress, nutrient, osmotic, humidity-transpiration imbalances, oxidative stress, and light stress are the forces generating these abnormalities [97]. Non-heritable genetic changes constitute some of the epigenetic changes, which are less stable, remain for a lesser period of time, and disappear on the cessation of stress condition [69]. DNA methylation, hypo- and hyperacetylation led some of the epigenetic changes occurring in in vitro-cultivated plant cells [25, 142]. Polycomb protein group modifies histone, and these proteins form conserve regulatory complexes that modify the chromatin state and gene expression during cellular transition from somatic to embryogenic cells. Two of such conserved regulatory complexes are Polycomb repressive complex 1 (PRC1) and PRC2. Trimethylation of histone 3 (H3K27me3) lysine 27 through SET-domain protein and subsequent binding of PRC1, which carry out ubiquitination of 119 lysine residues of histone H2A, improves compactness of the chromatin [109]. The state of chromatin determines binding of regulatory protein complexes and influences expression of genes.

In SV, the frequency of variations increases with the age of cultures, number of subcultures, and duration of stress [108]. The variations noted in plants regenerated through SE have both advantages and disadvantages. SV is a big problem where plants’ genetic and phenotypic integrity and purity are aimed at. In such cases, the genetic purity is ensured by taking the explants from authenticated, registered sources while the SV is also widely used in plant improvement programs [6]. The easily available variations among the regenerated plants could be profitable only when maintained stably for generations. The main problem of SV is the non-beneficial, redundant, and unstable variations, restricting the progress of breeding, and most of the regenerated plants showed poor agronomic performance [80, 81].

### Carbohydrates and underlying mechanism of SE

The reprogramming of signaling and communication of callus cells seem to be chemical in nature, and the analysis of callus exudates in the medium shows compounds like sugars, growth regulators, low molecular weight compounds, amino acids, and vitamins [16, 17]. Different carbohydrates were used as energy source in various media, of which sucrose and glucose are observed to be the most efficient for better cultural growth. In some plants, SE is absent until sucrose was added to the media, confirming its importance in embryo induction [75, 83]. For example, the expanded cotyledons of melon were noted to induce somatic embryos only in the presence of sucrose [52]. Sucrose or glucose may be substituted by other carbohydrates as carbon sources depending upon the tissue, plant, and species from which explants are taken [71]. Grzyb et al. [41] noted many fold effects of increased soluble sucrose at developmental transition to SE expression phase. Species-specific storage products are also accumulated during SE process and are absent in other stages of development [157].

### *Somatic Embryogenesis Receptor Kinase*, *SERK*, and other genes regulating SE

*SERK* is involved in embryogenic competence acquisition [152, 159]; the gene encodes protein and was isolated initially from carrot, named as *DcSERK*. Later, *SERK* homologues were also reported in many other plants (Table 1). Structurally, *SERK* consists of serine–proline-rich leucine zipper, kinase domain, signal peptide, leucine-rich region, transmembrane domain, and C-terminal region [152]. *SERK*, a cell surface receptor, triggers a signal cascade after binding to the ligand through the leucine-rich repeat (LRR) domain and with the help of intracellular domains reaches to the nucleus. This cascade alters gene expression pattern via chromatin remodelling [159]. Activity of genes is often altered either by repressing specific or selective genes and activating/changing the expression of others. *SERK* overexpression is observed during embryogenic induction till the globular stage and together with other genes like *BBM* and *LEC* promotes transition to embryogenic cells from non-embryogenic tissues [132].

**Table 1:** Genes/orthologs regulating somatic embryogenesis in various investigated plants

Genes/orthologs	Encoded products and possible role	Investigated plant	References
*ABI3* (*ABA INSENSITIVE 3*)	B2, B3 domain transcription factors; regulate embryo-specific ABA-inducible genes	*Arabidopsis thaliana*	Ikeda et al. [61]
*AGL15* (*AGAMOUS LIKE 15*)	MADS-box transcription factor; promote somatic embryogenesis	*Brassica napus*	Zhu and Perry [165]
*ASET1-3* (Alfalfa SE-specific transcripts)	Specific transcript, (product unknown); expressed at early stages of embryogenesis	*Medicago sativa*	Giroux and Pauls [39]
*AtECP31*, *AtECP63*	Embryogenic 31 and 63 cell proteins; expression during torpedo stage of embryogenesis, ABA-responsive genes	*A. thaliana*	Yang et al. [156]
*BBM* (*BABY BOOM*)	AP2/ERF Transcription factors; activates LEC1-ABI3-FUS3-LEC2 network to induce somatic embryogenesis	*B. napus*	Boutilier et al. [11] Hortsman [55]
*CaM* (Calmodulin genes)	Kinase type protein; accumulates during early embryogenesis through Ca-mediated signaling	Many plants	[5]
*Cdc2* (*Cell division cycle 2*)	Cdc protein; regulation of cell cycle progression	*M. sativa*	[96]
*CEM1*	Polypeptide, similar to translational elongation-factor 1α Expressed strongly pro-globular and globular stage	*Daucus carota*	[77]
*CGS102*, *CGS103*, *CGS201*	Glutamine synthetase; enzyme, expression during early SE stages	*D. carota*	[53]
*DcARG1* (*Auxin regulated Gene 1*)	Protein specific to auxin; expression at early induction stage	*D. carota*	[15]
*DcECP31*, *DcECP40*, *DcECP63*	Embryogenic cell protein; expression at torpedo stage of SE	*D. carota*	[15]
*FUS3* (*FUSCA 3*)	Transcriptional factor family protein; regulate synthesis of storage proteins and lipids	*A. thaliana*	[73]
*H3-1, H3-11* (Histone 3,11)	H3-1 gene transcript, auxin responsive	*M. sativa*	[74]
*Kryptonite* (*KYP/SUVH4*)	Methyl transferase; role in dedifferentiation	*A. thaliana*	[26]
*LATERAL ORGAN BOUNDARIES DOMAIN 29* (*LBD29*)	Transcription factor; dedifferentiation of cells, role in early embryogenesis	*A. thaliana*	[89]
*LEC1, LEC2* (*LEAFY COTYLEDON 1,2*)	B3 domain transcription factor; essential for somatic embryogenesis	*A. thaliana*	[21]
*PICKLE*	ATP-dependent chromatin remodeler; inhibits SE	*A. thaliana*	[120]
*PJCW1, PJCW2 = SAUR*, *SMALL AUXIN UP-REGULATED GENE*	Protein product, influence cell elongation	*Glycine max*	[45]
*POLYCOMB REPRESSIVE COMPLEX1* (*PRC 1*)	Epigenetic effector proteins; stem cell self-renewal, pluripotency, gene silencing; repressive effect on dedifferentiation ability of cells	*A. thaliana*	[26]
*PRIMORDIA TIMING*	Gene product; help in flower development; increases SAM cell population	*A. thaliana*	[49]
*SERK1-5* (*SOMATIC EMBRYO RECEPTOR KINASE 1-5*)	Receptor like kinase protein; acquisition of embryogenic competence	Many plants	[105]
*TOPI* (*Topoisomerase1*)	Constitutively expressed during cellular proliferative activities and at torpedo stage of SE development	*D. carota*	[7]
*WUSCHEL*	Homeo-domain transcription factor; Promote “vegetative to embryonic” transition	*A. thaliana*	[166]

*LEAFY COTYLEDON* (*LEC*) is one among the most important genes, playing a central role in both zygotic and somatic embryogeneses. Loss of functional mutation in *LEC* largely impaired the embryonic development [56]. The *LEC* mutant shows significantly reduced or total repression of embryogenic response as observed in double and triple mutants in *A. thaliana* [34]. The impairment is most ostensible in the maintenance of embryonic cell fate and specification of cotyledon identity. Overexpression of *LEC2* affects several target genes including the *AGAMOUS-like 15 (AGL15)* TF gene and auxin pathway genes [151]. *LEC2* mutants do not acquire desiccation tolerance and do not accumulate storage reserves in cotyledon tips [136]. Studies suggested that *FUSCA3* (*FUS3*), *LEC1*, and *LEC2* do not play a major role in the induction of SE, but during late stages of embryogenesis, their function has a significant say [56, 136]. Watery callus and root hairs are produced in *LEC1* single mutant, while *LEC1* and *FUS3* double and triple mutants negatively affect the SE process. Embryo identity and maturation are regulated by the network of LAFL proteins LEC1/LEC1-LIKE (L1L), ABSCISIC ACID INSENSITIVE 3 (ABI3), FUSCA3 (FUS3), and (LEC2) where B9 and B3 domains are encoded by *LEC1* and *LEC2* genes, respectively [145]. B9 is a subunit of NUCLEAR factor Y (NF-Y-B9), and B3 is a domain which contains transcription factor LEC2 [160] playing a role in maintaining the morphology of suspensor, progression via maturation phase, cotyledon identity specification, and suppressing premature germination [46]. Accumulation of storage macromolecules, desiccation tolerance, and cotyledon development are defective in zygotic embryos where loss of function mutation occurs in *LAFL* genes. LAFL proteins regulate the expression of BBM which gets reduced in case of *LAFL* mutant seeds [55]. *LEC2* have central role in maturation phase of SE; *LEC2* up regulates *AGL15* which is involved in the formation of somatic embryos from embryogenic tissues like zygotic embryos. *AGL15* and *LEC2* are involved in the activation of *INDOLE-3-ACETIC ACID INDUCIBLE 30* (*IAA30*) which when mutated affects the *AGL15*-mediated SE that normally shows enhancement under its effect [163]. Embryo development is switched on in the vegetative cells that acquire embryogenic competence under the influence of ectopic expression of *LEC* [29, 90, 137]. The *LEC* genes in turn seem to be regulated by *PICKLE* by causing chromatin remodelling, repressing the embryonic identity regulators during germination [84, 121].

*BABYBOOM* (*BBM*) is a transcription factor of AINTEGUMENTA-LIKE (AIL) APETALA2/ethylene-responsive element (AP2/ERF) family, isolated from *Brassica napus* embryos developed from pollen grains [11]. Ectopic expression of *BBM* in *A. thaliana* seedlings induces somatic embryos without the exogenous stress or growth regulator treatment. *BBM* along with other AP2/ERF family of transcription factors help in maintaining meristematic state of shoot and root meristems [56, 57]. It regulates cell growth and identity and promotes morphogenesis and cellular proliferation by exploiting AIL and LAFL proteins while mediating embryogenesis. Ectopic expression of *BBM* has an inductive effect in the formation of “somatic embryo-like structures” in *Arabidopsis*. *BBM* in SE binds to *YUCCA3* (*YUC3*), *YUC8*, and *TRYPTOPHAN AMINOTRANSFERASE OF ARABIDOPSIS1 (TAA1)* and promotes auxin biosynthesis, suggesting its role in endogenous auxin synthesis [151, 161]. *FUS3* and *LEC1* mutants completely abolish BBM-induced SE, suggesting their crucial role in *BBM*-induced SE pathway. Beside adventitious root, shoot formation, and SE induction, neoplastic growth (cell proliferation), deformed flowers, and leaves are the pleiotropic phenotypes of *BBM*. In *Theobroma cacao*, a higher level of *TcBBM* expression was noted during somatic embryogenesis than during zygotic embryogenesis time [30]. *BBM* also transcriptionally regulates *LEC*, *FUSCA3 (FUS3)*, and *ABI 45 INSENSITIVE3 (ABI3)* genes and induces cellular totipotency through LAFL network during seed germination [56]. *BBM* regulates the expression of *AGL15* and *LAFL* by binding to promoter of genes. This is evident from the observation where *AGL15* and *LEC2* mutants show reduced *BBM*-mediated SE.

Other genes like *LATE EMBRYO ABUNDANT (LEA)* are noted to be abundantly expressed during later phases of embryogenesis [107]. The LEA proteins are hydrophilic and are regulated by ABA [60]. The LEA proteins influence the developmental processes of zygotic and somatic embryogeneses and also to stress-related responses. In almost all instances, their expression is observed in embryogenic tissue and not in vegetative cells. In addition to LEA proteins, some other genes like *WUSCHEL* are active during SE; *WUS* develops somatic embryos indirectly, and ectopic expression of *WUS* also produces somatic embryo directly and promotes organogenesis on exogenous auxin-amended or PGR-free cultures as evidenced in *WUS* mutants [88]. The emergence of shoots forming embryos similarly occurs in ectopically expressed *WUS* explants in auxin-free and *CLAVATA (CLV)* mutants in 2,4-D (auxin)–added medium [164]. *WUS* and *CLV* normally function to maintain stem cells and cell differentiation in shoot meristem [166]. Cell differentiation is also regulated by these genes in the shoot apical meristem (SAM) of *CLV* mutants where somatic embryos are formed by some non-committed cells [61, 166]. *WOUND INDUCED DEDIFFERENTIATION1 (WIND1)* or RAP2-4 (Protein RELATED to APETALA2 4) induces SE and play a role in callus formation in tissue damage and wounding [63]. *PLETHORA2 (PLT2)* plays a major role in the induction and specification of root pole in SE [11, 146]. Reverse glycosylating protein (RGP-1), a membrane protein, encourages plant cell wall development by facilitating polysaccharide metabolism, and in early phases of somatic embryogenesis, it is thought to participate in structural reorganization [37]. *AGAMOUS-like 15* (*AGL 15***)** is isolated as a MADS-box gene, detected in many plants (e.g., *B. napus*, *Arabidopsis*, *Taraxacum*), and in alfalfa, it is detected in somatic embryos [60]. *AGL15* regulates the expression of several genes during the process of SE by encoding MADS-box family of transcription factors. For example, AtGA2ox6 is encoded by a gene, controlled by *AGL15* [60]. Overexpression of *AGL15* induces SE in embryogenic tissue like zygotic embryos and could not induce SE spontaneously in *Arabidopsis* seedlings. Ectopic expression of *AGL15* under CaMV35S promoter induces embryo formation in seedling in which 2,4-D and *AGL15* both regulate expression [165].

**Fig. 2:**
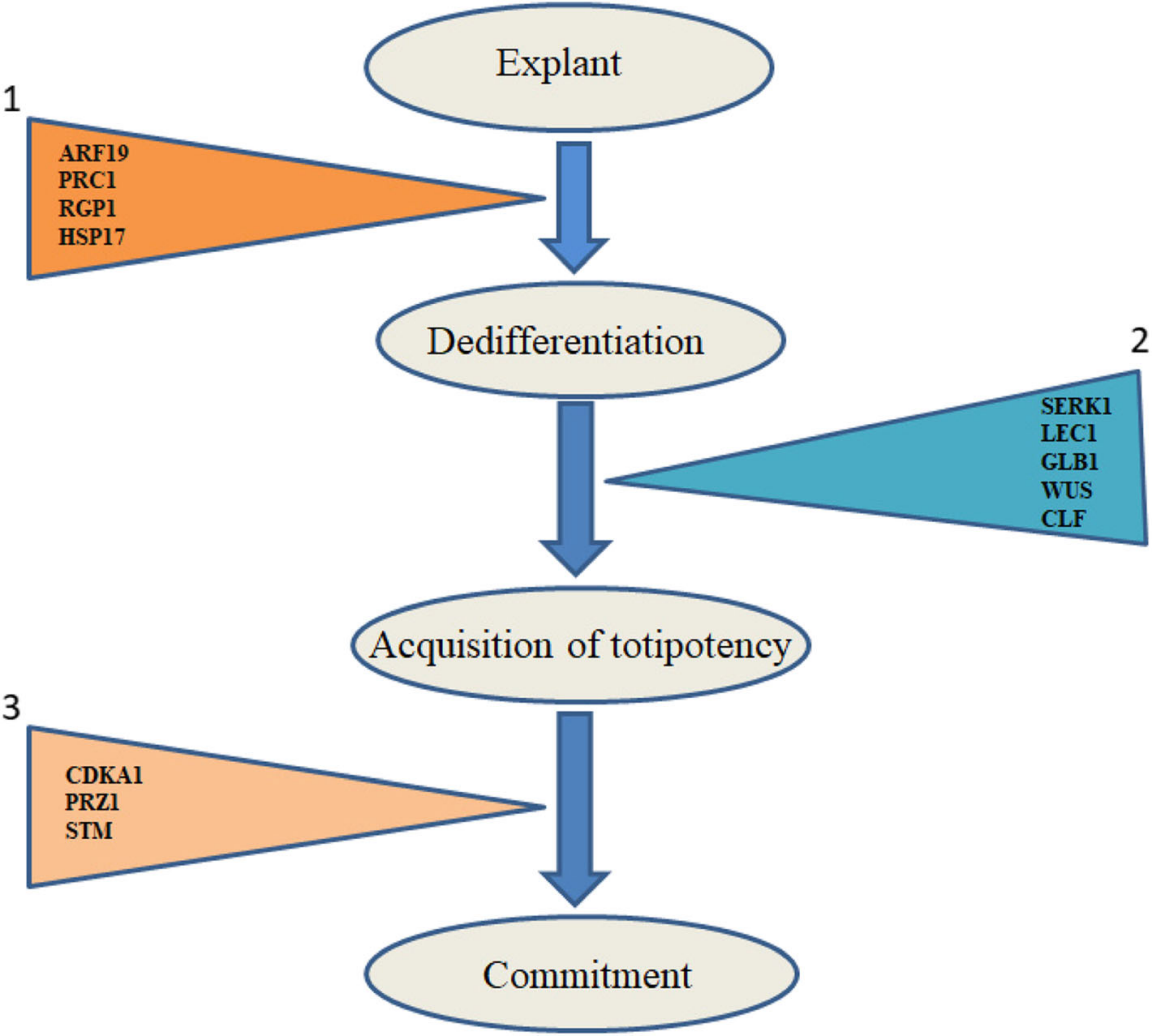
*Different genes at different stages of SE pathway. Triangle 1 in yellow shows genes involved in dedifferentiation; triangle 2 shows genes involved in acquisition of totipotency by the cells; and triangle 3 shows genes expressed in commitment of totipotent cells to embryogenic state. AUXIN RESPONSE FACTOR 19 (ARF19), POLYCOMB REPRESSIVE COMPLEX 1 (PRC1), REVERSIBILY GLYCOSYLATED POLYPEPTIDE 1 (RGP1), HEAT SHOCK PROTEIN 17 (HSP17), SOMATIC EMBRYOGENESIS LIKE RECEPTOR KINASE (SERK1), LEAFY COTYLEDON1 (LEC1), GALACTOSIDASE BETA 1 (GLB1), WUSCHEL (WUS), CURLY LEAF (CLF), CYCLIN DEPENDENT KINASE A1 (CDKA1), PROPORZ1 (PRZ1), SHOOT MERISTEMLESS (STM)*

Among the different RKD (RWP-RK domain-containing) proteins, only RWP-RK DOMAIN-CONTAINING 4 (RKD4) is noted to produce embryos; RWP-RK DOMAIN-CONTAINING 4 (RKD4)/GROUNDED (GRD) also induces embryos and is thought to be expressed in maximum in suspensors and early stages of embryos [57]. On the overexpression of *RKD4,* SE develops into seedlings by stimulating root cells to proliferate; and in *RKD4* mutants, embryo development is arrested, and suspensor remains short [55]. Different genes/transcription factors (TFs) playing various roles at different stages of embryogenesis are shown in Fig. 2.

The mystery behind the SE is being gradually unfolded by the use of molecular approach. Over 700 TFs and genes are being extensively studied during the process of SE in *Arabidopsis thaliana* and other plants, suggesting the very significant role of TF in competence acquisition via embryogenic reprogramming [40]. Some of the genes and TFs having a role in SE are enlisted in Table 2. Studies suggest that the basic mechanism behind the somatic and zygotic embryogenesis is the same, and the genes regulating zygotic embryogenesis have very similar effect on SE. Differentially expressed genes *DEG1* and *DEG2* associated with embryogenesis were identified in *Dactylis glomerata* [3]; *DEG*s express in the embryogenic leaf (not in non-embryogenic cells) and is noted in both directly and indirectly induced cultures, while *DEG2* expression is noted only in directly induced tissues. The ectopic expression of various zygotic embryogenic genes significantly increased the somatic embryo development in several investigated plants. Similarly, the chromatin remodeling determines spatial and temporal expression of genes and influences the development of SE to a large extent [4]. Indirect SE requires more extensive chromatin modification than that of direct SE as was shown by differential expression of chromatin modifiers after 2,4-D–mediated callus formation [23]

**Table 2:** *SOMATIC EMBRYOGENESIS RECEPTOR KINASE* (*SERK*) gene regulating embryogenesis in different studied plant materials

Name of plant	Common name	*SERK* gene	References
*Adiantum capillus-veneris*	Maidenhair fern	*AcvSERK*	[87]
*Ananas comosus*	Pineapple	*Ac SERK1–3*	[91]
*Arabidopsis thaliana*	Thale cress	*At SERK1–5*	[47]
*Citrus sinensis*	Orange	*Cs SERK*	[38]
*Citrus unshiu*	Tangerine	*Cu SERK*	[131]
*Cocos nucifera*	Coconut	*Cn SERK*	[114]
*Cucurma alismatifolia*	Summer tulip	*CaSERK*	[139]
*Cyclamen persicum*	Persian cyclamen	*Cp SERK1–2*	[128]
*Cyrtochilum loxense*	Not available	*Cl SERK*	[19]
*Dactylis glomerata*	Orchard grass	*DgSERK*	[134]
*Daucus carota*	Carrot	*Dc SERK*	[129]
*Dimocarpus longan*	Longan	*Dl SERK*	[1]
*Garcinia mangostana*	Magnosteen	*Mangosteen SERK*	[122]
*Glycine max*	Soya bean	*Gm SERK1–2*	[155]
*Gossypium hirsutum*	Cotton	*Gh SERK1–3*	[111]
*Helianthus annuus*	Sunflower	*HaSERK*	[143]
*Marchantia polymorpha*	Common liverwort	*Mp SERK*	[127]
*Medicago truncatula*	Barrel clover	*Mt SERK1–6*	[105]
*Musa acuminata*	Banana	*MaSERK*	[59]
*Nicotiana benthamiana*	Tobacco	*Nb SERK3A*, *Nb SERK3B*	[93]
*Ocotea catharinensis*	Not available	*OcoteaSERK*	[125]
*Oryza sativa*	Rice	*OsbiSERK*, *Os SERK*, *Os SERKlike1*, *Os SERKlike2*	[66]
*Physcomitrella patens*	Moss	*Pp SERK1–3*	[1]
*Poa pratensis*	Common meadow grass	*Poap SERKlike1–2*	[2]
*Populus trichocarpa*	Black cottonwood	*Pp SERK1-4*	Aan den Toorn et al. [1]
*Prunus persica*	Peach	Persica SERK*	[67]
*Prunus salicina*	Japenese plum	*PsSERK*	[67]
*Rosa canina*	Dog rose	*RcSERK*	x [78]
*Rosa hybrid*	Hybrid tea rose	*RhSERK1–4*	[158]
*Selaginella moellendorffii*	Club moss	*Sm SERK1–4*	[1]
*Solanum lycopersicum*	Tomato	*Sl SERK1*, *Sl SERK3A*, *Sl SERK3B*	[93]
*Solanum peruvianum*	Wild tomato	*Sp SERK*	[1]
*Solanum tuberosum*	Potato	*St SERK*	[130]
*Sorghum bicolor*	Sorghum	*Sb SERK1–3*	[1]
*Theobroma cacao*	Cocoa tree	*TcSERK*	[126]
*Triticum aestivum*	Wheat	*Ta SERK1*, *Ta SERK2*, *Ta SERKlike3*	Singla et al. [133]
*Vitis vinifera*	Grape	*Vv SERK1–3*	[92]
*Zea mays*	Maize	*Zm SERK1–3*	[8]

Modified and courtesy: [141]

### SE-related proteins

Currently, a novel combination of techniques is being utilized for the identification and quantification of embryo-specific proteins, which cannot otherwise be identified by conventional gel-based methodologies. Liquid chromatography–mass spectroscopy (LC–MS) is a technique in which liquid chromatography and mass spectroscopy operate together and in tandem. In this technique, the protein sample is processed/digested into small fragments and separated after loading in the LC column; and subsequent analysis is made based on mass/charge ratio (m/z). The technique is used for the identification of proteins using different softwares like SEQUEST, MASCOT, and Proteome discoverer. Helleboid [48] reported glucanases, chitinases, and osmotin-like proteins (also called pathogen-related or PR proteins) which accumulate during SE of *Cichorium*. These and other similar proteins were isolated from different plants including tobacco during the hypersensitive reactions against the tobacco mosaic virus, classified into five major groups PR1–PR5. Later, it was established that such proteins accumulate during stress conditions like injury, heavy metals, plant hormones, and UV. Similarly, other SE-related proteins were reported in different plants [e.g., *Zea mays* [35], *Araucaria angustifolia* [31], *Coffea arabica* [12], *Picea asperata* [70], *Gossypium hirsutum* [36], *Larix principis-rupprechtii* [162], *Picea balfouriana* [85], *Saccharum* spp. [50], and *Catharanthus roseus* [43]]. One class of 14-3-3 proteins play a significant role in plant immunity, cell cycle control, metabolism, stress responses, transcription, signal transduction, programmed cell death protein trafficking, and SE [106]. These are acidic regulatory proteins, binding in a phosphorylation-dependent manner to target proteins like phosphothreonine and phosphoserine and thus have a significant role in plant growth and development. Heat shock proteins, peroxidase, catalase, superoxide dismutase, etc. are some other proteins that are common in many plants, accumulate in SE tissues, and are studied via gel-free shotgun proteomics. Several proteins isolated during SE are stress proteins suggesting that stressed microenvironment is the driving force for SE induction. Of these different proteins, several were identified as proteomic markers. The most common proteins identified as potential markers of SE are listed in Table 3.

**Table 3:** Plants and different SE related proteins, identified through LC-MS

Some important SE-related proteins	Plant/species	References
Alcohol dehydrogenase, allene oxide synthase, ATP synthase, glyceraldehyde-3-phosphate dehydrogenase, GH3 protein, glutathione-S transferases, heat shock proteins, indole-3-acetic acid-amidosynthetase, late embryogenesis abundant, lipid transfer protein, peroxidase, photosystem II proteins, ribosomal proteins, ribulose-1,5 bisphosphate carboxylase, superoxide dismutase, sucrose synthase	*Gossypium hirsutum*	[36]
14-3-3 protein, 6-phosphogluconate dehydrogenase, actin, aldose 1-epimerase, annexin, ADP-ribosylation factor GTPase-activating proteins, ATP synthase, calmodulin, catalase, chitinase, citrate synthase, clathrin, elongation factors, eukaryotic initiation factors, glyceraldehyde-3-phosphate dehydrogenase, glycine-rich RNA-binding proteins, heat shock cognate proteins, histones, heat shock proteins, importin, superoxide dismutase, triosephosphateisomerase, tubulin, peroxidase, ubiquitin	*Larix principis-rupprechtii*	[162]
14-3-3 protein, actin, aldose 1-epimerase, annexin, ATP synthase, ADP-ribosylation factor GTPase-activating proteins, calmodulin, chitinase, citrate synthase, glycine-rich RNA-binding proteins, heat shock cognate proteins, heat shock proteins, importin, peroxidase, triosephosphateisomerase, tubulin	*Larix principis-rupprechtii*	[162]
Calmodulin, germin-like proteins, glutathione-S transferases, peroxidase, ribosomal proteins, superoxide dismutase	*Picea balfouriana*	[85]
Actin, aldolase, catalase, germin-like proteins, late embryogenesis abundant, secreted protein, tubulin	*Saccharum* spp.	[50]
14-3-3 proteins, actin, alcohol dehydrogenase, ATP synthase, chitinase, elongation factors, glyceraldehyde-3 phosphate dehydrogenase, glutathione-S transferases, histones, heat shock proteins, PIN-like protein, ribulose-1,5-bisphosphate carboxylase, ubiquitin	*Araucaria angustifolia*	[31]
Aldolase, chitinase, glyceraldehyde-3-phosphate dehydrogenase, peroxidase	*Coffea arabica*	[12]
14-3-3 proteins, arabinogalactan proteins, glutathione-S transferases, heat shock proteins, indole-3-acetic acid-amidosynthetase, late embryogenesis abundant, peroxidase, ubiquitin	*Saccharum* spp.	[119]
Alcohol dehydrogenase, aldose 1-epimerase, allene oxide synthase, catalase, chitinase, glutathione-S transferases, heat shock proteins, indole-3-acid-amidosynthetase, late embryogenesis abundant, peroxidase, photosystem II proteins, ribosomal proteins, ribulose-1,5-bisphosphate carboxylase, sucrose synthase, tubulin	*Picea asperata*	[70]
6-phosphogluconate dehydrogenase, annexin, clathrin, eIFs, histones, heat shock proteins, lipid transfer protein, peroxidase, ribosomal proteins	*Saccharum* spp	[51]
14-3-3 proteins, chitinase, GH3 protein, glutathione-S transferases, indole-3-acetic acid-amidosynthetase, peroxidase, tubulin	*Zea mays*	[35]
14-3-3 proteins, chitinase, GH3 protein, glutathione-S transferases, peroxidase, tubulin, annexin, clathrin, eIFs, histones, heat shock proteins, late embryogenesis abundant, chitinase, PR proteins, importin, catalase, etc.	*Catharanthus roseus*	[43]

## Conclusions

Since the first report of SE, this intricate process has been studied extensively in a large number of plant genera of dicots, monocots, gymnosperms, and fern. Various stages of embryogenesis (i.e., embryo origin, development, maturation, and germination into plantlets) have also been unveiled. The factors controlling somatic embryogenesis have also been identified; some of them are plant genotype, explant, medium composition, carbohydrate type, oxygen concentration, PGRs, and various stresses. Although the molecular mechanism is still not well elucidated, chromatin remodeling, activation and deactivation of genes, and complicated transcription networks are linked with somatic and zygotic embryogenesis processes. A number of genes or orthologs which have important say in early cellular transition from somatic to embryogenic cells are *AUXIN RESPONSE FACTORs, POLYCOMB REPRESSIVE COMPLEX 1* (*PRC1*), *REVERSIBILY GLYCOSYLATED POLYPEPTIDE 1* (*RGP1*), and *HEAT SHOCK PROTEIN 17* (*HSP17*), *SOMATIC EMBRYOGENESIS LIKE RECEPTOR KINASE* (*SERK1*), *LEAFY COTYLEDON1* (*LEC1*), *WUSCHEL* (*WUS*), *CURLY LEAF* (*CLF*). The expression of *SHOOT MERISTEMLESS* (*STM*) gene influences in other stages of somatic embryogenesis. Several proteins may act as potential markers for the process of SE (e.g., 14-3-3 protein, chitinase, LEA, etc.). At the time of genetically uniform plant propagation, genetic transformation, artificial seed production, plant regeneration from protoplast, and in biodiversity conservation, the SE information will be very indispensable. Flow cytometry, nano LC–MS, real-time PCR, and other sensitive molecular techniques have a scope in understanding the molecular mechanism underlying SE. These may refine the process, scale up the progress of research in SE, and may increase its application in other novel fields.

## References

[1] Aan den Toorn M, Albrecht C, de Vries S (2015). On the origin of SERKs: bioinformatics analysis of the somatic embryogenesis receptor kinases. Mol Plant.

[2] Albertini E, Marconi G, Reale L, Barcaccia G, Porceddu A, Ferranti F, Falcinelli M (2005). *SERK* and *APOSTART*: candidate genes for apomixis in *Poa pratensis*. Plant Physiol.

[3] Alexandrova K, Conger B (2002). Isolation of two somatic embryogenesis-related genes from orchard grass (*Dactylis glomerata*). Plant Sci.

[4] Altamura MM, Della Rovere F, Fattorini L, D'Angeli S, Falasca G (2016). Recent advances on genetic and physiological bases of *in vitro* somatic embryo formation. Methods Mol Biol.

[5] Anil VS, Harmon AC, Sankara Rao K (2000). Spatio-temporal accumulation and activity of calcium-dependent protein kinases during embryogenesis, seed development, and germination in sandalwood. Plant Physiol.

[6] Bahmankar M, Mortazavian S MM, Tohidfar M, Sadat Noori SA, Izadi Darbandi A, Corrado G, Rao R (2017) Chemical compositions, somatic embryogenesis and somaclonal variation in cumin. Bio Med Res Int 1-15 10.1155/2017/7283806 PMC5694991 29234682

[7] Balestrazzi A, Toscano I, Bernacchia G, Luo M, Otte S, Carbonera D (1996). Cloning of a cDNA encoding DNA topoisomerase I in *Daucus carota* and expression analysis in relation to proliferation. Gene.

[8] Baudino S, Hansen S, Brettshneider R, Hecht VFG, Dresselhaus T, Lors H, Dumas C, Rogowsky PM (2001). Molecular characterization of two novel maize LRR receptor-like kinase, which belong to the *SERK* gene family. Planta.

[9] Beena MR, Winter S, Makeshkumar T (2016). Influence of age of explants and genotype on somatic embryogenesis in African and Indian cassava cultivars. J Root Crops.

[10] Boulard C, Fatihi A, Lepiniec L, Dubreucq B (2017). Regulation and evolution of the interaction of the seed B3 transcription factors with NF-Y subunits. Biochimica et Biophysica Acta.

[11] Boutilier K, Offringa R, Sharma VK, Kieft H, Ouellet T, Zhang L, Hattori J, Liu CM, van Lammeren AA, Miki BL, Custers JB, van LookerenCampagne MM (2002). Ectopic expression of BABY BOOM triggers a conversion from vegetative to embryonic growth. Plant Cell.

[12] Campos NA, Paiva LV, Panis B, Carpentier SC (2016). The proteome profile of embryogenic cell suspensions of *Coffea arabica* L. Proteomics.

[13] Campos NA, Panis B, Carpentier SC (2017). Somatic embryogenesis in coffee: the evolution of biotechnology and the integration of omics technologies offer great opportunities. Frontiers Plant Sci.

[14] Chiancone B, Germanà MA (2013). Micropropagation of *Citrus* spp. by organogenesis and somatic embryogenesis. Protocols for micropropagation of selected economically-important horticultural plants.

[15] Chugh A, Khurana P (2002). Gene expression during somatic embryogenesis - recent advances. Curr Sci.

[16] Chung JP, Chang TL, Chi AYM, Shi CT (2006). Triploid banana cell growth phases and the correlation of medium pH changes with somatic embryogenesis in embryogenic cell suspension culture. Plant Cell Tissue Org Cult.

[17] Chung JP, Lu CC, Kuo LT, Ma SS, Shi CT (2016). Acidogenic growth model of embryogenic cell suspension culture and qualitative mass production of somatic embryos from triploid bananas. Plant Cell Tissue Org Cult.

[18] Corredoira E, Ballester A, Ibarra M, Vieitez AM (2015). Induction of somatic embryogenesis in explants of shoot cultures established from adult *Eucalyptus globulus* and *E. saligna x E. maidenii* trees. Tree Physiol.

[19] Cueva A, Concia L, Cella R (2012). Molecular characterization of a *Cyrtochilum loxense* Somatic Embryogenesis Receptor-like Kinase (*SERK*) gene expressed during somatic embryogenesis. Plant Cell Rep.

[20] Cullis MA, Swennen R, Cullis CA (2007). Genomic changes associated with somaclonal variation in banana (Musa spp.). Physiol Plant.

[21] Curaba J, Moritz T, Blervaque R, Parcy F, Raz V, Herzog M, Vachon G (2004). *AtGA3ox2*, a key gene responsible for bioactive gibberellin biosynthesis, is regulated during embryogenesis by *LEAFY COTYLEDON2* and *FUSCA3* in *Arabidopsis*. Plant Physiol.

[22] De Feria M, Jimenez E, Barbon R, Capote A, Chavez M, Quiala E (2003). Effect of dissolved oxygen concentration on differentiation of somatic embryos of *Coffea arabica* cv. Catimor 9722. Plant Cell. Tissue Org Cult.

[23] De-la-Pena C, Nic-Can GI, Galaz-Avalos RM, Avilez-Montalvo R, Loyola-Vargas VM (2015). The role of chromatin modifications in somatic embryogenesis in plants. Front Plant Sci.

[24] Delporte F, Pretova A, du Jardin P, Watillon B (2014). Morpho-histology and genotype dependence of *in vitro* morphogenesis in mature embryo cultures of wheat. Protoplasma.

[25] Duarte-Ake F, Castillo-Castro E, Pool FB, Espadas F, Santamaria JM, Robert ML, De-la-Pena C (2016). Physiological differences and changes in global DNA methylation levels in *Agave angustifolia* Haw. albino variant somaclones during the micropropagation process. Plant Cell Rep.

[26] Elhiti M, Stasolla C, Wang A (2013). Molecular regulation of plant somatic embryogenesis. In Vitro Cell Develop Biol –Plant.

[27] Elmeer KES, Junaid A, Srivastava PS, Sharma MP (2013). Factors regulating somatic embryogenesis in plants. Somatic embryogenesis and gene expression.

[28] Feher A (2015). Somatic embryogenesis - stress-induced remodeling of plant cell fate. Biochimica et Biophysica Acta.

[29] Feher A, Pasternak TP, Dudits D (2003). Transition of somatic plant cells to an embryogenic state. Plant Cell, Tissue Org Cult.

[30] Florez SL, Erwin RL, Maximova SN, Guiltinan MJ, Curtis WR (2015). Enhanced somatic embryogenesis in *Theobroma cacao* using the homologous BABY BOOM transcription factor. BMC Plant Biol.

[31] Fraga HP, Vieria LN, Heringer AS, Puttkammer CC, Silveira V, Guerra MP (2016). DNA methylation and proteome profiles of *Araucaria angustfolia* (Bertol) Kuntze embryogenic cultures as affected by plant growth regulators supplementation. Plant Cell Tissue Org Cult.

[32] Fuentes SRL, Calheiros MBP, Manetti-Filho J, Vieira LGE (2000). The effects of silver nitrate and different carbohydrate sources on somatic embryogenesis in *Coffea canephora*. Plant Cell Tissue Org Cult.

[33] Fujimura T (2014). Carrot somatic embryogenesis. A dream come true. Plant Biotechnol Rep.

[34] Gaj MD, Zhang S, Harada JJ, Lemaux PG (2005). Leafy cotyledon genes are essential for induction of somatic embryogenesis of *Arabidopsis*. Planta.

[35] Ge F, Hu H, Huang X, Zhang Y, Wang Y, Li Z, Zou C, Peng H, Li L, Gao S, Pan G, Shen Y (2017). Metabolomic and proteomic analysis of maize embryonic callus induced from immature embryo. Sci Rep.

[36] Ge X, Zhang C, Wang Q, Yang Z, Wang Y, Zhang X, Wu Z, Hou Y, Wu J, Li F (2015). iTRAQ protein profile differential analysis between somatic globular and cotyledonary embryos reveals stress, hormone, and respiration involved in increasing plantlet regeneration of *Gossypium hirsutum* L. J Proteome Res.

[37] Ge XX, Chai LJ, Liu Z, Wu XM, Deng XX, Guo WW (2012). Transcriptional profiling of genes involved in embryogenic, non-embryogenic calluses and somatic embryogenesis of Valencia sweet orange by SSH-based microarray. Planta.

[38] Ge XX, Fan GE, Chai L, Guo WW (2010). Cloning, molecular characterization and expression analysis of a SOMATIC EMBRYOGENESIS RECEPTOR-LIKE KINASE gene (CitSERK1-like) in Valencia sweet orange. Acta Physiol Plant.

[39] Giroux RW, Pauls KP (1997). Characterization of somatic embryogenesis-related cDNAs from alfalfa (*Medicago sativa* L.). Plant Mol Biol.

[40] Gliwicka M, Nowak K, Balazadeh S, Mueller-Roeber B, Gaj MD (2013). Extensive modulation of the transcription factor transcriptome during somatic embryogenesis in *Arabidopsis thaliana*. PloS One.

[41] Grzyb M, Kalandyk A, Waligórski P, Mikula A (2017). The content of endogenous hormones an sugars in the process of early somatic embryogenesis in the tree fern *Cyathea delgadii* Sternb. Plant Cell Tissue Org Cult.

[42] Guan Y, Li SG, Fan XF, Su ZH (2016). Application of somatic embryogenesis in woody plants. Front Plant Sci.

[43] Gulzar B, Mujib A, Rajam MV, Frukh A, Zafar N (2019) Identification of somatic embryogenesis (SE) related proteins through label-free shotgun proteomic method and cellular role in Catharanthus roseus (L.) G. Don. Plant Cell Tiss. and Org. Cult. 10.1007/s11240-019-01563-0

[44] Guo F, Liu C, Xia H, Bi Y, Zhao C, Zhao S, Hou L, Li F, Wang X (2013). Induced expression of AtLEC1 and AtLEC2 differentially promotes somatic embryogenesis in transgenic tobacco plants. PloS One.

[45] Hagen G, Kleinschmidt A, Guilfoyle T (1984). Auxin-regulated gene expression in intact soybean hypocotyl and excised hypocotyl sections. Planta.

[46] Han JD, Li X, Jiang CK, Wong GK, Rothfels CJ, Rao GY (2017). Evolutionary analysis of the LAFL genes involved in the land plant seed maturation program. Front Plant Sci.

[47] Hecht V, Vielle-Calzada JP, Hartog MV, Schmidt EDL, Boutilier K, Grossniklaus U (2001). The *Arabidopsis* somatic embryogenesis receptor kinase 1 gene is expressed in developing ovules and embryos and enhances embryogenic competence in culture. Plant Physiol.

[48] Helleboid S, Hendriks T, Bauw G, Inze D, Vasseur J, Hilbert JL (2000). Three major somatic embryogenesis related proteins in *Cichorium* identified as PR proteins. J Exp Bot.

[49] Helliwell CA, Chin-Atkins AN, Wilson IW, Chapple R, Dennis ES, Chaudhury A (2001). The *Arabidopsis* AMP1 gene encodes a putative glutamate carboxypeptidase. Plant Cell.

[50] Heringer AS, Barroso T, Macedo AF, Santa-Catarina C, Souza GHMF, Floh EIS, Souza-Filho GA, Silveira V (2015) Label-free quantitative proteomics of embryogenic and non-embryogenic callus during sugarcane somatic embryogenesis. Plos One. 10.1371/journal.pone.0127803 10.1371/journal.pone.0127803 PMC4452777 26035435

[51] Heringer AS, Reis RS, Passaman LZ, de Souza-Filho GA, Santa-Catarina C, Silveira V (2017). Comparative proteomics analysis of the effect of combined red and blue lights on sugarcane somatic embryogenesis. Acta Physiol. Plant.

[52] Hideki N, Takeshi S, Naoki Y, Masayoshi S, Shunji K, Akiko I (2001). Effects of sugars and abscisic acid on somatic embryogenesis from melon (Cucumis melo L.) expanded cotyledon. Sci Hort.

[53] Higashi K, Shiota H, Kamada H (1998). Patterns of expression of the genes for glutamine synthetase isoforms during somatic and zygotic embryogenesis in carrot. Plant Cell Physiol.

[54] Hofmann N (2014). Getting to the root of regeneration: adventitious rooting and callus formation. The Plant Cell.

[55] Horstman A, Bemer M, Boutilier KA (2017). Transcriptional view on somatic embryogenesis. Regeneration.

[56] Horstman A, Li M, Heidmann I, Weemen M, Chen B, Muino JM, Angenent GC, Boutilier K (2017). The BABY BOOM transcription factor activates the LEC1-ABI3-FUS3-LEC2 network to induce somatic embryogenesis. Plant Physiol.

[57] Horstman A, Willemsen V, Boutilier K, Heidstra R (2014). AINTEGUMENTA-LIKE proteins: hubs in a plethora of networks. Trends Plant Sci.

[58] Hu R, Sun Y, Wu B, Duan H, Zheng H, Hu D, Lin H, Tong Z, Xu J, Li Y (2017). Somatic embryogenesis of immature *Cunninghamia lanceolata* (lamb.) hook zygotic embryos. Sci Rep.

[59] Huang X, Lu XY, Zhao JT, Chen JK, Dai XM, Xiao W, Chen YP, Chen YF, Huang XL (2010). *MaSERK1* gene expression associated with somatic embryogenic competence and disease resistance response in banana (*Musa* spp.). Plant Mol Biol Rep.

[60] Ikeda M, Kamada H (2005). Comparison of molecular mechanisms of somatic and zygotic embryogenesis. Plant Cell Monogr.

[61] Ikeda M, Umehara M, Kamada H (2006). Embryogenesis-related genes; its expression and roles during somatic and zygotic embryogenesis in carrot and *Arabidopsis*. Plant Biotechnol.

[62] Ikeuchi M, Ogawa Y, Iwase A, Sugimoto K (2016). Plant regeneration: cellular origins and molecular mechanisms. Development.

[63] Ikeuchi M, Sugimoto K, Iwase A (2013). Plant callus: mechanisms of induction and repression. Plant Cell.

[64] Indoliya Y, Tiwari P, Chauhan AS, Goel R, Shri M, Bag SK, Chakrabarty D (2016). Decoding regulatory landscape of somatic embryogenesis reveals differential regulatory networks between japonica and indica rice subspecies. Sci Rep.

[65] Isah T (2016). Induction of somatic embryogenesis in woody plants. Acta Physiol Plant.

[66] Ito Y, Takaya K, Kurata N (2005). Expression of *SERK* family receptor-like protein kinase genes in rice. Biochim Biophys Acta.

[67] Jayanthi M, Jerard A, Sherif S, Jayasankar S (2014). Molecular characterization of somatic embryogenesis receptor-like kinase(SERK) genes from plum (*Prunus salicina)* and peach (*Prunus persica)*. Ind Jhortic.

[68] Jiménez VM (2001). Regulation of *in vitro* somatic embryogenesis with emphasis on the role of endogenous hormones. Revista Brasileira de Fisiologia Vegetal.

[69] Jin S, Mushke R, Zhu H, Tu L, Lin Z, Zhang Y, Zhang X (2008). Detection of somaclonal variation of cotton (*Gossypium hirsutum*) using cytogenetics, flow cytometry and molecular markers. Plant Cell Rep.

[70] Jing D, Zhang J, Xia Y, Kong L, OuYang F, Zhang S, Zhang H, Wang J (2016) Proteomic analysis of stress-related proteins and metabolic pathways in *Picea asperata* somatic embryos during partial desiccation. Plant Biotechnol J doi. 10.1111/pbi.12588 10.1111/pbi.12588 PMC5253475 27271942

[71] Junaid A, Mujib A, Fatima S, Sharma MP (2008). Cultural conditions affect somatic embryogenesis in *Catharanthus roseus* L. (G.) Don. Plant Biotechnol Rep.

[72] Junker A, Monke G, Rutten T, Keilwagen J, Seifert M (2012). Elongation-related functions of *LEAFY COTYLEDON1* during the development of *Arabidopsis thaliana*. Plant J.

[73] Kagaya Y, Toyoshima R, Okuda R, Usui H, Yamamoto A, Hattori T (2005). LEAFY COTYLEDON1 controls seed storage protein genes through its regulation of FUSCA3 and ABSCISIC ACID INSENSITIVE3. Plant Cell Physiol.

[74] Kapros T, Bogre L, Nemeth K, Bako L, Gyorgyey J, Wu SC, Denes Dudits D (1992). Differential expression of Histone H3 gene variants during cell cycle and somatic embryogenesis in alfalfa. Plant Physiol.

[75] Karami O, Aghavaisi B, Pour AM (2009). Molecular aspects of somatic-to-embryogenic transition in plants. J Chem Biol.

[76] Karami O, Deljou A, Esna-Ashari M, Ostad-Ahmadi P (2006). Effect of sucrose concentrations on somatic embryogenesis in carnation (*Dianthus caryophyllus* L.). Sci Hort.

[77] Kawahara R, Sunabori S, Fukuda H, Komamlne A (1992). A gene expressed preferentially in the globular stage of somatic embryogenesis encodes elongation-factor Ia in carrot. Eur J Biochem.

[78] Kedong X, Qinglin L, Huifang Y, Li Z, Lili D, Fengluan L, Ling B, Nan M, Liangiun Z (2011). Isolation and molecular characterization of RcSERK1: a *Rosa canina* gene transcriptionally induced during initiation of protocorm-like bodies. Afr J Biotechnol.

[79] Kocak M, Izgu T, Sevindik B, Tutuncu M, Curuk P, Simsek O, Kacar YA, Teixeira da Silva JA, Mendi YY (2014). Somatic embryogenesis of Turkish *Cylamen persicum* Mill. Sci Hort.

[80] Kokina I, Mickevica I, Jermalonoka M, Bankovska L, Gerbreders V, Ogurcovs A, Jahundovica I (2017) Case study of somaclonal variation in resistance genes Mlo and Pme3 in Flaxseed (*Linum usitatissimum* L.) induced by nanoparticles. Inter J Genomics. 10.1155/2017/1676874 10.1155/2017/1676874 PMC5343275 28326314

[81] Krishna H, Alizadeh M, Singh D, Singh U, Chauhan N, Eftekhari M, Sadh RK (2016) Somaclonal variations and their applications in horticultural crops improvement. 3 Biotech 6: 54 doi: 10.1007/s13205-016-0389-7 10.1007/s13205-016-0389-7 PMC4752953 28330124

[82] Lelu-Walter MA, Thompson D, Harvengt L, Sanchez L, Toribio M, Pâques LE (2013). Somatic embryogenesis in forestry with a focus on Europe: state-of-the-art, benefits, challenges and future direction. Tree Genet Genomes.

[83] Lema-Ruminska J, Goncerzewicz K, Gabriel M (2013) Influence of abscisic acid and sucrose on somatic embryogenesis in Cactus *Copiapoa tenuissima* Ritt. *forma mostruosa*. The Sci World J. 10.1155/2013/513985 10.1155/2013/513985 PMC3694557 23843737

[84] Li HC, Chuang K, Henderson JT, Rider SD, Bai Y, Zhang H, Fountain M, Gerber J, Ogas J (2005). PICKLE acts during germination to repress expression of embryonic traits. The Plant J.

[85] Li Q, Zhang S, Wang J (2015). Transcriptomic and proteomic analyses of embryogenic tissues in *Picea balfouriana* treated with 6-benzylaminopurine. Physiol Plant.

[86] Li SB, Xie ZZ, Hu CG, Zhang JZ (2016). A review of Auxin Response Factors (ARFs) in plants. Front Plant Sci..

[87] Li X, Fang YH, Han JD, Bai SN, Rao GY (2014) Isolation and characterization of a novel somatic embryogenesis receptor kinase gene expressed in the fern *Adiantum capillus-veneris* during shoot regeneration *in vitro*. Plant Mol Biol Rep doi. 10.1007/s11105-014-0769-2

[88] Liang Y, Xiong Z, Zheng J (2016) Genome-wide identification, structural analysis and new insights into late embryogenesis abundant (LEA) gene family formation pattern in *Brassica napus*. Sci Rep 6. 10.1038/srep 10.1038/srep24265 PMC4829847 27072743

[89] Liu HI, Wang GC, Feng Z, Zhu J (2010). Screening of genes associated with dedifferentiation and effect of LBD29 on pericycle cells in *Arabidopsis thaliana*. Plant Growth Reg.

[90] Lotan T, Ohto M, Yee KM, West MA, Lo R, Kwong RW, Yamagishi K, Fischer RL, Goldberg RB, Harada JJ (1998). Arabidopsis *LEAFY COTYLEDON1* is sufficient to induce embryo development in vegetative cells. Cell.

[91] Ma J, He Y, Hu Z, Xu W, Xia J, Guo C, Lin S, Cao L, Chen C, Wu C, Zhang J (2012). Characterization and expression analysis of AcSERK2, a somatic embryogenesis and stress resistance related gene in pineapple. Gene.

[92] Maillot P, Lebel S, Schellenbaum P, Jacques A, Walter B (2009). Differential regulation of *SERK*, *LEC*-like and pathogenesis related genes during indirect secondary somatic embryogenesis in grapevine. Plant Physiol Biochem.

[93] Mantelin S, Peng HC, Li B, Atamian HS, Takken FL, Kaloshian I (2011). The receptor-like kinase SlSERK1 is required for Mi-1-mediated resistance to potato aphids in tomato. Plant J.

[94] Marquez-Lopez RE, Perez-Hernandez C, Ku-Gonzalez A, Galaz-Avalos RM, Loyola-Vargas VM (2017) Localization and transport of indole-3-acetic acid during somatic embryogenesis in *Coffea canephora*. Protoplasma. 10.1007/s00709-017-1181-1 10.1007/s00709-017-1181-1 29119309

[95] Merkle SA, Dean JF (2000). Forest tree biotechnology. Curr Opin Biotechnol.

[96] Mészáros T, Miskolczi P, Ayaydin F, Pettko-Szandtner A, Peres A, Magyar Z, Horvath GV, Bako L, Feher A, Dudits D (2000). Multiple cyclin-dependent kinase complexes and phosphatases control G2/M progression in alfalfa cells. Plant Mol Biol.

[97] Miguel C, Marum L (2011). An epigenetic view of plant cells cultured *in vitro*: somaclonal variation and beyond. J Exp Bot.

[98] Mikula A, Pozoga M, Tomiczak K, Rybczynski JJ (2015). Somatic embryogenesis in ferns: a new experimental system. Plant Cell Rep.

[99] Morcillo F, Gagneur C, Adam H, Richaud F, Singh R, Cheah SC, Rival A, Duval Y, Tregear JW (2006). Somaclonal variation in micropropagated oil palm Characterization of two novel genes with enhanced expression in epigenetically abnormal cell lines and in response to auxin. Tree physiol.

[100] Mozgova I, Munoz-Viana R, Hennig L (2017). PRC2 represses hormone-induced somatic embryogenesis in vegetative tissue of *Arabidopsis thaliana*. PLoS Genetics.

[101] Mujib A (2016) Somatic embryogenesis in ornamentals and its applications. Springer, p 267. 10.1007/978-81-322-2683-3

[102] Mujib A, Ali M, Tonk D, Isah T, Zafar N (2016) Embryogenesis in ornamental monocots: plant growth regulators as signaling element. In. A. Mujib (ed). Somatic embryogenesis in ornamentals and its application. Springer, pp. 187- 201

[103] Mujib A, Samaj J (2006). Somatic embryogenesis.

[104] Naing AH, Kim CK, Yun BJ, Jin JY, Lim KB (2013). Primary and secondary somatic embryogenesis in *Chrysanthemum c*v. Euro. Plant Cell Tissue Org Cult.

[105] Nolan KE, Irwanto RR, Rose RJ (2003). Auxin up-regulates *MtSERK1* expression in both *Medicago truncatula* root-forming and embryogenic cultures. Plant Physiol..

[106] Oh CS (2010). Characteristics of 14-3-3 proteins and their role in plant immunity. Plant Pathol J.

[107] Olvera-Carrillo Y, Campos F, Reyes JL, Garciarrubio A, Covarrubias AA (2010). Functional analysis of the group 4 late embryogenesis abundant proteins reveals their relevance in the adaptive response during water deficit in Arabidopsis. Plant Physiol.

[108] Ong-Abdullah M, Ordway JM, Jiang N, Ooi SE, Kok SY, Sarpan N, Azimi N, Hashim AT, Ishak Z, Rosli SK, Malike FA, Bakar NA, Marjuni M, Abdullah N, Yaakub Z, Amiruddin MD, Nookiah R, Singh R, Low ET, Chan KL, Azizi N, Smith SW, Bacher B, Budiman MA, Van Brunt A, Wischmeyer C, Beil M, Hogan M, Lakey N, Lim CC, Arulandoo X, Wong CK, Choo CN, Wong WC, Kwan YY, Alwee SS, Sambanthamurthi R, Martienssen RA (2015). Loss of Karma transposon methylation underlies the mantled somaclonal variant of oil palm. Nature.

[109] Orłowska A, Igielski R, Łagowska K, Kępczyńska E (2017). Identification of *LEC1*, *L1L* and Polycomb Repressive Complex 2 genes and their expression during the induction phase of *Medicago truncatula* Gaertn. somatic embryogenesis. Plant Cell, Tiss Org Cult.

[110] Ozudogru EA, Lambardi M (2016). Cryotechniques for the long-term conservation of embryogenic cultures from woody plants. In: *In vitro* embryogenesis in higher plants. Methods Mol Biol.

[111] Pandey DK, Chaudhary B (2014). Oxidative stress responsive SERK1 gene directs the progression of somatic embryogenesis in cotton (*Gossypium hirsutum* L. cv. Coker 310). Amer J Pl Sci.

[112] Pasternak TP, Prinsen E, Ayaydin F, Miskolczi P, Potters G, Asard H, Van Onckelen HA, Dudits D, Feher A (2002). The role of auxin, pH, and stress in the activation of embryogenic cell division in leaf protoplast-derived cells of alfalfa. Plant Physiol.

[113] Pencik A, Tureková V, Paulisiç S, Rolcìk J, Strnad M, Mihaljevic S (2015). Ammonium regulates embryogenic potential in *Cucurbita pepo* through pH-mediated changes in endogenous auxin and abscisic acid. Plant Cell Tissue Organ Cult.

[114] Pérez-Núñez MT, Souza R, Sáenz L, Chan JL, Zúñiga-Aguilar JJ, Oropeza C (2009). Detection of a *SERK*-like gene in coconut and analysis of its expression during the formation of embryogenic callus and somatic embryos. Plant Cell Rep.

[115] Pulianmackal AJ, Kareem AV, Durgaprasad K, Trivedi ZB, Prasad K (2014). Competence and regulatory interactions during regeneration in plants. Front Plant Sci.

[116] Quiroz-Figueroa FR, Rojas-Herrera R, Galaz-Avalos RM, Loyola-Vargas VM (2006). Embryo production through somatic embryogenesis can be used to study cell differentiation in plants. Plant Cell Tissue Org Cult.

[117] Raemakers K, Pereira I, Koehorst van Putten H, Visser R (2006) Indirect somatic embryogenesis in cassava for genetic modification purposes. In: Loyola-Vargas VM, Vázquez-Flota, F, (Eds.) Methods Mol Biol 318: 101–109 10.1385/1-59259-959-1:101 16673909

[118] Raghavan V (2006). Can carrot and Arabidopsis serve as model systems to study the molecular biology of somatic embryogenesis?. Curr Sci.

[119] Reis RS, Vale EM, Heringer AS, Santa-Catarina C, Silveira V (2016). Putrescine induces somatic embryo development and proteomic changes in embryogenic callus of sugarcane. J Proteom.

[120] Rider SD, Hemm MR, Hostetler HA, Li HC, Chapple C, Ogas J (2004). Metabolic profiling of the Arabidopsis pkl mutant reveals selective derepression of embryonic traits. Planta.

[121] Rider SD, Henderson JT, Jerome RE, Edenberg HJ, Romero-Severson J, Ogas J (2003). Coordinate repression of regulators of embryonic identity by PICKLE during germination in *Arabidopsis*. Plant J.

[122] Rohani ER, Ismanizan I, Noor NM (2012). Somatic embryogenesis of mangosteen. Plant Cell Tiss Organ Cult.

[123] Rutledge RG, Stewart D, Overton C, Klimaszewska K (2017). Gene expression analysis of primordial shoot explants collected from mature white spruce (*Picea glauca*) trees that differ in their responsiveness to somatic embryogenesis induction. PloS One.

[124] Sané D, Aberlenc-Bertossi F, Diatta LID, Guèye B, Daher A, Sagna M, Borgel A (2012) Influence of growth regulators on callogenesis and somatic embryo development in date palm (*Phoenix dactylifera* L.) Sahelian Cultivars. The Scientific World J. 10.1100/2012/837395 10.1100/2012/837395 PMC3353711 22629211

[125] Santa-Catarina C, Hanai LR, Dornelas MC, Viana AM, Floh EIS (2004). *SERK* gene homolog expression, polyamines and amino acids associated with somatic embryogenic competence of *Ocotea catharinensis* Mez. (Lauraceae). Plant Cell Tiss Organ Cult.

[126] Santos MO, Romano E, Yotoko KSC, Tinoco MLP, Dias BBA, Aragão FJL (2005). Characterisation of the cacao somatic embryogenesis receptor-like kinase (SERK) gene expressed during somatic embryogenesis. Plant Sci.

[127] Sasaki G, Katoh K, Hirose N, Suga H, Kuma K, Miyata T, Su ZH (2007). Multiple receptor-like kinase cDNAs from liverwort *Marchantia polymorpha* and two charophycean green algae, *Closterium ehrenbergii* and *Nitella axillaris:* extensive gene duplications and gene shufflings in the early evolution of streptophytes. Gene.

[128] Savona M, Mattioli R, Nigro S, Falasca G, Della Rovere F, Costantino P, De Vries SC, Ruffoni B, Trovato M, Altamura MM (2012). Two SERK genes are markers of pluripotency in *Cyclamen persicum* Mill. J Exp Bot.

[129] Schmidt ED, Guzzo F, Toonen MA, de Vries SC (1997). A leucine rich repeat containing receptor-like kinase marks somatic plant cells competent to form embryos. Development.

[130] Sharma SK, Millam S, Hein I, Bryan GJ (2008). Cloning and molecular characterisation of potato *SERK* gene transcriptionally induced during initiation of somatic embryogenesis. Planta.

[131] Shimada T, Hirabayashi T, Endo T, Fujii H, Kita M, Omura M (2005). Isolation and characterization of the somatic embryogenesis receptor-like kinase gene homologue, (*CitSERK1*) from *Citrus unshiu* Marc. Sci Hort.

[132] Singh A, Khurana P (2017). Ectopic expression of *Triticum aestivum* SERK genes (TaSERKs) control plant growth and development in *Arabidopsis*. Sci Rep.

[133] Singla B, Khurana JP, Khurana P (2008). Characterization of three somatic embryogenesis receptor kinase genes from wheat, *Triticum aestivum*. Plant Cell Rep.

[134] Somleva MN, Schmidt EDL, de Vries SC (2000). Embryogenic cells in *Dactylis glomerata* L. (Poaceae) explants identified by cell tracking and by *SERK* expression. Plant Cell Rep.

[135] Stasolla C, Yeung EC (2003). Recent advances in conifer somatic embryogenesis: improving somatic embryo quality. Plant Cell Tissue Org Cult.

[136] Stone SL, Braybrook SA, Paula SL, Kwong LW, Meuser J, Pelletier J, Hsieh TF, Fischer RL, Goldberg RB, Harada JJ (2008). Arabidopsis LEAFY COTYLEDON2 induces maturation traits and auxin activity: implications for somatic embryogenesis. Proceed Nat Acad Sci.

[137] Stone SL, Kwong LW, Yee KM, Pelletier J, Lepiniec L, Fischer RL, Goldberg RB, Harada JJ (2001). LEAFY COTYLEDON2 encodes B3 domain transcription factor that induces embryo development. Pro Natl Acad Sci.

[138] Su YH, Zhao XY, Liu YB, Zhang CL, O'Neill SD, Zhang XS (2009). Auxin-induced WUS expression is essential for embryonic stem cell renewal during somatic embryogenesis in *Arabidopsis*. The Plant J.

[139] Sucharitakul K, Rakmit R, Boonsorn Y, LeelaponO TT, Bunnag S, Chanvivattana Y (2014) Isolation and expression analysis of a SOMATIC EMBRYOGENESIS RECEPTOR-LIKE KINASE(SERK) gene in Curcuma alistatifolia Gagnep. J Agric Sci doi. 10.5539/jas.v6n10p207

[140] Tagipur ME, Seker G, Teixeira da Silva JA, Mendi YY, Mujib A (2016). Somatic embryogenesis, cryopreservation, and *in vitro* mutagenesis in *Cyclamen*. Somatic embryogenesis in ornamentals and its applications.

[141] Talapatra S, Goswami P, Das S, Raychaudhuri SS, Mujib A (2016). Role of SERK during somatic embryogenesis and its interaction with brassinosteroids. Somatic embryogenesis in ornamentals and its applications.

[142] Testillano PS, Risueno MC (2016). Detection of epigenetic modifications during microspore embryogenesis: analysis of DNA methylation patterns dynamics. Methods Mol Biol.

[143] Thomas C, Meyer D, Himber C, Steinmetz A (2004). Spatial expression of a sunflower *SERK* gene during induction of somatic embryogenesis and shoot organogenesis. Plant Physiol Biochem.

[144] Thorpe T (2012). History of plant tissue culture. Methods Mol Biol.

[145] Valon C, Savino G, Guilleminot J, Devic M, Giraudat J, Parcy F, To A (2006). A network of local and redundant gene regulation governs *Arabidopsis* seed maturation. The Plant Cell.

[146] Tsuwamoto R, Yokoi S, Takahata Y (2010). Arabidopsis EMBRYOMAKER encoding an AP2 domain transcription factor plays a key role in developmental change from vegetative to embryonic phase. Plant Mol Biol.

[147] Verdeil JL, Alemanno L, Niemenak N, Tranbarger TJ (2007). Pluripotent versus totipotent plant stem cells: dependence versus autonomy?. Trends Plant Sci.

[148] Vieitez AM, Barciela J (1990). Somatic embryogenesis and plant regeneration from embryonic tissues of *Camellia japonica* L. Plant Cell Tissue Org Cult.

[149] von Arnold S, Sabala I, Bozhkov P, Dyachok J, Filonova L (2002). Developmental pathways of somatic embryogenesis. Plant Cell Tissue Organ Cult.

[150] Wojcikowska B, Gaj MD (2017). Expression profiling of AUXIN RESPONSE FACTOR genes during somatic embryogenesis induction in *Arabidopsis*. Plant Cell Rep.

[151] Wojcikowska B, Jaskola K, Gasiorek P, Meus M, Nowak K, Gaj MD (2013). LEAFY COTYLEDON2 (LEC2) promotes embryogenic induction in somatic tissues of Arabidopsis, via YUCCA-mediated auxin biosynthesis. Planta.

[152] Wu W, Wu Y, Gao Y, Li M, Yin H, Lv M, Zhao J, Li J, He K (2015). Somatic embryogenesis receptor-like kinase 5 in the ecotype Landsberg erecta of *Arabidopsis* is a functional RD LRR-RLK in regulating brassinosteroid signaling and cell death control. Frontiers Plant Sci.

[153] Wu XB, Wang J, Liu JH, Deng XX (2009). Involvement of polyamine biosynthesis in somatic embryogenesis of Valencia sweet orange (*Citrus sinensis*) induced by glycerol. J Plant Physiol.

[154] Yaacob JS, Taha RM, Esmaeili AK (2013) Comparative studies on cellular behaviour of carnation (*Dianthus caryophyllus* Linn. cv. Grenadin) grown *in vivo* and *in vitro* for early detection of somaclonal variation. The SciWorld J. 10.1155/2013/686752 10.1155/2013/686752 PMC3674743 23766703

[155] Yang C, Zhao T, Yu D, Gai J (2011). Isolation and functional characterization of a SERK gene from soybean (*Glycine max* (L.) Merr.). Plant Mol Biol Rep.

[156] Yang H, Saitou T, Komeda Y, Harada H, Kamada H (1997). *Arabidopsis thaliana ECP63* encoding a LEA protein is located in chromosome 4. Gene.

[157] Yang X, Zhang X (2010). Regulation of somatic embryogenesis in higher plants. Crit Rev Plant Sci.

[158] Zakizadeh H, Stummann BM, Lutken H, Muller R (2010). Isolation and characterization of four somatic embryogenesis receptor-like kinase (*RhSERK*) genes from miniature potted rose (*Rosa hybrida* cv. Linda). Plant Cell Tissue Organ cult.

[159] Zhang H, Lin X, Han Z, Wang J, Qu LJ, Chai J (2016). SERK family receptor-like kinases function as co-receptors with PXY for plant vascular development. Mol Plant.

[160] Zhang Y, Clemens A, Maximova SN, Guiltinan MJ (2014). The *Theobroma cacao* B3 domain transcription factor TcLEC2 plays a duel role in control of embryo development and maturation. BMC Plant Biol.

[161] Zhao Y (2014). Auxin biosynthesis. The Arabidopsis Book/American Society of Plant Biologists.

[162] Zhao J, Li H, Fu S, Chen B, Sun W, Zhang J (2015) AniTRAQ-based proteomics approach to clarify the molecular physiology of somatic embryo development in Prince Rupprecht’s larch (*Larix principis-rupprechtii* Mayr). PloSOne 10.1371/journal.pone.0119987. eCollection 2015 10.1371/journal.pone.0119987 PMC4363690 25781987

[163] Zheng Y, Ren N, Wang H, Stromberg AJ, Perry SE (2009). Global identification of targets of the Arabidopsis MADS domain protein AGAMOUS-Like15. The Plant Cell.

[164] Zhou X, Zheng R, Liu G, Xu Y, Zhou Y, Laux T, Zhen Y, Harding SA, Shi J, Chen J (2017). Desiccation treatment and endogenous IAA levels are key factors influencing high frequency somatic embryogenesis in *Cunninghamia lanceolata* (Lamb.) Hook. Front. Plant Sci.

[165] Zhu C, Perry SE (2005). Control of expression and autoregulation of AGL15, a member of the MADS-box family. The Plant J.

[166] Zuo J, Niu Q-W, Frugis G, Chua N-H (2002). The *WUSCHEL* gene promotes vegetative-to-embryonic transition in *Arabidopsis*. The Plant J.

